# Effect of Aeration Rate Redistribution on Nitrogen Removal Performance of a Novel Multi-Compartment Fixed-Biofilm Cyclic Activated Sludge System

**DOI:** 10.3390/microorganisms14051099

**Published:** 2026-05-13

**Authors:** Zichun Yan, Shuichao Fan, Wankai Yan, Haopeng Ma, Tianhao Zhao

**Affiliations:** 1 School of Environmental and Municipal Engineering, Lanzhou Jiaotong University, Lanzhou 730070, China; fanshuichao317@outlook.com (S.F.); yanwaikai2002@outlook.com (W.Y.); mahaopeng2026@outlook.com (H.M.); 20230208127@stu.lzjtu.edu.cn (T.Z.); 2 Key Laboratory of Yellow River Water Environment of Gansu Province, Lanzhou 730070, China

**Keywords:** biological nitrogen removal, fixed-biofilm cyclic activated sludge system, aeration rate redistribution, microbial community structure

## Abstract

To address the problems of short-circuit flow and dead zones, complicated operation and control caused by intermittent influent, and the mismatch between aeration rate and oxygen demand in the Cyclic Activated Sludge System (CASS), a novel Multi-Compartment Fixed-Biofilm Cyclic Activated Sludge System (MCFCASS) was developed. This system operated in continuous-flow mode, and the aeration rate of each compartment was redistributed using a mathematical model. The results show that the plug flow ratio of the MCFCASS reactor increased from 18.75% to 31.25% compared with the CASS reactor. After aeration rate redistribution, the average total nitrogen (TN) removal efficiency of the MCFCASS reactor rose from 83.34% to 86.80%, and the effluent TN concentration consistently met the Grade I-A limit (15 mg/L) specified in the Discharge Standard of Pollutants for Municipal Wastewater Treatment Plant (GB 18918-2002). The average removal efficiencies of chemical oxygen demand (COD) and ammonium nitrogen (NH_4_^+^-N) increased from 91.58% and 93.39% to 92.98% and 94.57%, respectively. Microbial community analysis revealed that after aeration rate redistribution, the relative abundances of Pseudomonadota, Bacteroidota, and Bacillota in the pre-reaction zone of MCFCASS were 39.17%, 17.78%, and 10.33%, respectively. In addition, the abundances of some functional bacterial groups in the first and fourth compartments of the main reaction zone shifted adaptively in response to the aeration rate redistribution, consistent with the trends in pollutant removal contributions in these compartments. Hierarchical clustering and principal coordinate analysis (PCoA) further indicated that aeration rate redistribution influenced the microbial community structure. The above laboratory-scale optimization results may provide a preliminary reference for aeration control and improvement of denitrification performance in similar processes.

## 1. Introduction

With rapid global urbanization and population growth, wastewater discharge has increased annually. The discharge of nitrogen-containing pollutants into water bodies tends to cause eutrophication [1,2]. At present, wastewater treatment plants are facing the challenge of economically and efficiently removing nitrogen from wastewater to meet increasingly stringent discharge standards [3].

The Cyclic Activated Sludge System (CASS) is a modified Sequencing Batch Reactor (SBR) process. The CASS reactor, as the core unit of the process, combines the advantages of a biological selector and a sequencing batch activated sludge reactor [4]. Because of its low construction cost and maintenance cost, this process has been widely applied in municipal and various industrial wastewater treatment plants, especially for smaller facilities in China [5]. However, the conventional CASS process suffers from poor hydraulic conditions such as short-circuit flow and dead zones in practical operation [5]. Poor flow patterns often lead to insufficient contact between bacteria and nitrogen, which adversely affects the treatment capability of the process [6,7]. In addition, compared with continuous-flow reactors, intermittent influent reactors are more complex in operation and control [8].

Dead zones and short-circuit flow frequently occur in practical bioreactors due to reactor structure, flow distribution, and mixing conditions, impairing pollutant removal efficiency [6,9,10]. Relevant studies have demonstrated that dividing a reactor into multiple compartments with baffles can effectively reduce the dead zone ratio, increase the plug flow proportion in the reactor, and improve pollutant removal efficiency [11,12,13]. Ghalekhani compared the performance of a conventional activated sludge membrane bioreactor (AS-MBR) and a compartmentalized activated sludge membrane bioreactor (CAS-MBR) for industrial park wastewater treatment, reporting that the removal efficiency of CAS-MBR exceeded 98%, higher than the maximum COD removal efficiency of 94% achieved by AS-MBR [14].

Aeration mode is also a key factor affecting pollutant removal performance [15]. For plug flow reactors, the pollutant concentration decreases along the reactor, leading to a mismatch between the uniform aeration mode and the oxygen demand in the front and rear sections [16,17]. Chen et al. conducted comparative experiments with different aeration modes for municipal wastewater and found that the tapered aeration group (R2) achieved a TN removal efficiency of 76.25%, higher than the 69.35% achieved by the uniform aeration group (R1) [18]. Jeong et al. employed a combined process of a rotating biological contactor and tapered aeration reactors to treat wastewater discharged from a food factory, and the system achieved COD and TN removal efficiencies of 98% and 93%, respectively [19]. Zhang et al. found that gradually reduced aeration optimized the dominant microbial community and its function in the aerobic granular sludge process, with the Shannon diversity index increased to 5.79 and the TN removal efficiency reaching approximately 85% [20].

Meanwhile, for anoxic/oxic (A/O) denitrification processes, reducing the dissolved oxygen (DO) concentration in the recirculated mixed liquor benefits denitrification process in the anoxic tank [21]. Urbini et al. found that adding an anoxic reaction zone after the aerobic nitrification stage reduced the DO concentration in the mixed liquor returned to the front pre-reaction zone, increasing the denitrification efficiency to 91% [22]. Liu et al. employed intermittent aeration in a Modified Ludzack–Ettinger (MLE) process, which lowered the DO concentration in the internal recirculation mixed liquor and created alternating anoxic/anaerobic conditions in the front mixing zone, thereby improving TN removal by approximately 20% when treating conventional municipal wastewater [23].

To address the above problems, this study developed a novel Multi-Compartment Fixed-Biofilm Cyclic Activated Sludge System (MCFCASS), aiming to reduce the dead zone ratio, increase the plug flow proportion, and optimize the aeration mode to match the aeration rate with oxygen demand in each compartment. Furthermore, the system operated in continuous-flow mode, which simplifies operation and control.

## 2. Materials and Methods

### 2.1. Domestic Sewage and Seed Sludge

The experimental wastewater was collected from the domestic sewage on the campus of Lanzhou Jiaotong University. During the experimental period, the water quality parameters were as follows: NH_4_^+^-N 53.27–71.74 mg/L, COD 300.5–379.9 mg/L, and TN 63.78–103.79 mg/L. The seed sludge was obtained from Lanzhou Xigu Wastewater Treatment Plant, and the inoculum volume was 10 L.

### 2.2. Experimental Setup

**Figure 1:**
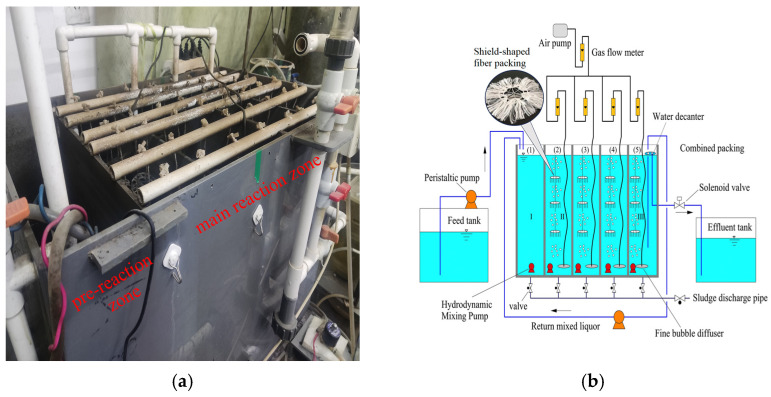
Experimental setup: (**a**) Photograph of the MCFCASS reactor; (**b**) Schematic diagram of the MCFCASS reactor.

As shown in Figure 1a, the MCFCASS reactor consisted of a pre-reaction zone and a main reaction zone. As shown in Figure 1b, the first compartment along the influent direction served as the pre-reaction zone, with an effective volume of 44 L, corresponding to the position labeled (1) in the schematic diagram. The subsequent four compartments, (2), (3), (4), and (5), constituted the main reaction zone, each with an effective volume of 44 L. The pre-reaction zone was hydraulically mixed by a submersible pump (WP-2990; from Weierma, Guangzhou, China) to achieve mixing of sludge and wastewater and prevent sludge deposition. The main reaction zone was aerated using fine bubble diffusers (ϕ92 mm, 8 units; from Yuanrun, Gongyi, China) with high oxygen transfer efficiency and fine bubbles to supply oxygen efficiently, and was supplemented by submersible pumps (WP-2990, 4 units) for mixing. Shield-shaped fiber packing (Yilan, Yixing, China) with a specific surface area of 1300 m^2^/m^3^ was suspended in each compartment of the main reaction zone. The packing consists of a polypropylene support ring, on which polyvinyl alcohol (PVA) fiber bundles are evenly distributed. As shown in Figure 1b, the sampling locations of suspended sludge in the pre-reaction zone, the first and fourth compartments of the main reaction zone were marked as I, II, and III, respectively. Suspended sludge samples were collected from the middle height of each compartment, and biofilm samples were collected at a height of 10 cm from the bottom. As a control for the flow pattern experiment, the CASS reactor also consisted of a pre-reaction zone and a main reaction zone. The pre-reaction zone had an effective volume of 44 L, while the main reaction zone, which is not divided into compartments, had an effective volume of 176 L. The aeration and mixing modes were the same as those of the MCFCASS reactor.

### 2.3. Experimental and Analytical Methods

#### 2.3.1. Water Quality Analysis and Determination Methods

NH_4_^+^-N and TN were determined using Nessler’s reagent spectrophotometry and alkaline potassium persulfate digestion UV spectrophotometry, respectively [24]. NH_4_^+^-N was measured with a 721G visible spectrophotometer (INESA, Shanghai, China). COD was determined using the potassium dichromate method [25]. BOD (Biochemical Oxygen Demand) was determined by the dilution and seeding method [26]. TN and COD were determined using a HACH DR5000 UV–visible spectrophotometer (HACH, Loveland, CO, USA). Dissolved oxygen (DO) was measured using a HACH HQ10 meter (HACH, Loveland, CO, USA).

#### 2.3.2. Microbial Analysis Methods

In this study, the most probable number (MPN) method was used to cultivate and enumerate denitrifying bacteria in the pre-reaction zone of the MCFCASS reactor [27].

In this study, 16S rRNA high-throughput sequencing was applied to analyze the bacterial community structure in the reactor system [28]. Total microbial genomic DNA was extracted from all samples using the E.Z.N.A.^®^ Soil DNA Kit (Omega Bio-tek, Norcross, GA, USA) in accordance with the manufacturer’s instructions. The V3–V4 hypervariable regions of the bacterial 16S rRNA gene were amplified with the primer set 338F (5′-ACTCCTACGGGAGGCAGCAG-3′) and 806R (5′-GGATACHVGGGTWTCTAAT-3′). Paired-end sequencing was conducted on an Illumina PE300/PE250 platform (Illumina, San Diego, CA, USA) according to the standard protocols of Majorbio Bio-Pharm Technology Co., Ltd. (Shanghai, China).

Under tapered aeration, the first and fourth compartments of the main reaction zone in the MCFCASS reactor, which exhibited the greatest difference in microbial community, were selected as sampling areas. Before aeration rate redistribution, the suspended activated sludge samples collected from the first and fourth compartments of the main reaction zone were labeled as AS_1b and AS_4b, and the corresponding biofilm samples were designated as BS_1b and BS_4b, respectively. After aeration rate redistribution, the suspended activated sludge sample from the pre-reaction zone was denoted as AS_p; the suspended activated sludge samples from the first and fourth compartments of the main reaction zone were labeled as AS_1a and AS_4a, and the associated biofilm samples were marked as BS_1a and BS_4a, respectively.

## 3. Results

### 3.1. Optimization of Aeration Rate Redistribution in the MCFCASS Reactor

As the organic matter concentration gradually decreased along the MCFCASS reactor, a mathematical model was established based on hydraulic experiments to allocate the aeration rate of each compartment on demand.

#### 3.1.1. Characteristics of Hydraulic Flow Pattern in the MCFCASS Reactor

The two-compartment CASS reactor was dominated by a completely mixed flow pattern. By dividing into multiple compartments, the plug flow proportion in the MCFCASS reactor may be increased [29]. The flow patterns of the CASS and MCFCASS reactors were analyzed using hydraulic flow pattern experiments. The CASS reactor and the MCFCASS reactor were regarded as a series combination of plug flow and completely mixed flow, and its residence time distribution function can be expressed as follows [30]:

(1) $$\left\{\begin{array}{c} \;E\left(t\right)\;=\;\frac{Q}{V}\exp\frac{-Q}{V}\left[\;\;t\;-\;\left(1\;-\;f_{1}\right)\frac{V}{Q}\right]\;t\;>\;\left(1\;-\;f_{1}\right)\frac{V}{Q}\\ E\left(t\right)\;=\;0\;\;\;\;0\;<\;t\;\leq \;\left(1\;-\;f_{1}\right)\frac{V}{Q} \end{array}\right.,$$

The relationship between the critical time and the fraction of completely mixed flow is:

(2) $$t_{c}\;=\;\left(1\;-\;f_{1}\right)\frac{V}{Q},$$

where f_1_ is the fraction of completely mixed flow (%), Q is the effluent flow rate of the reactor (L/min), V is the reactor volume (L), and t_c_ is the critical time (min) approximately determined by the peak time of conductivity.

The pulse tracer method was employed to conduct flow pattern experiments [31]. The aeration rate of both reactors was 0.8 m^3^/h, the influent flow rate was 0.183 L/min, and the effective reactor volume was 220 L. Sodium chloride (NaCl) was used as the tracer. A saturated solution prepared from 100 g of NaCl was rapidly injected into the turbulent zone of the influent. The conductivity at the outlets of each individual compartment and of the entire reactor was continuously monitored using a conductivity meter. A non-uniform sampling interval was adopted: the first sample was collected at 5 min after tracer injection, followed by sampling at 10 min intervals from 5 to 75 min and at 30 min intervals from 75 to 1815 min. The total monitoring duration was 1815 min to ensure complete capture of the rising limb, peak, and falling limb of the residence time distribution (RTD) curve. Three independent replicate tracer experiments were conducted, and the average results of the replicate experiments were taken as the final measured values.

The fraction of completely mixed flow f_1_ can be calculated by Equation (2), and the results are presented in Table 1.

As shown in Table 1, the fraction of plug flow in the MCFCASS reactor was 31.25%, which was considerably higher than the 18.75% in the CASS reactor. Meanwhile, the fraction of completely mixed flow in a single compartment of the MCFCASS reactor was 85.42%. The configuration of the MCFCASS reactor ensured sufficient mixing and mass transfer in individual compartments, while making the overall flow pattern closer to plug flow relative to the CASS reactor, thus effectively restraining back-mixing and short-circuiting [32,33,34].

The actual mean residence time ($\bar{t}$) of the MCFCASS reactor was 776.71 min, which can be obtained by Equation (3).

(3) $$\overset{-}{t}\;\approx \;\frac{\sum \left(t_{i+1}+t_{i}\right)\left(\rho_{i+1}+\rho_{i}\right)\left(t_{i+1}\;-\;t_{i}\right)}{2\sum \left(\rho_{i+1}+\rho_{i}\right)\left(t_{i+1}\;-\;t_{i}\right)},$$

where ρ_i_ is the tracer concentration at time t_i_ (g/L), t_i+1_ − t_i_ is the time interval between consecutive tracer samples (min), and t_i_ is the sampling time of the i-th sample (min).

#### 3.1.2. Redistribution of Aeration Rate in the MCFCASS Reactor

To match the aeration rate with the oxygen demand in each compartment of the main reaction zone in the MCFCASS reactor, a mathematical model for aeration rate redistribution was established in this study using the following procedures. To facilitate model derivation, the following assumptions were made: the degradation of BOD in domestic sewage follows first-order reaction kinetics; the flow pattern in each compartment is a combination of completely mixed flow and plug flow; the effluent from the preceding compartment serves as the influent to the subsequent compartment; the oxygen demand per kg of BOD removed ($a^{\prime }$) and the oxygen demand per kg of mixed liquor volatile suspended solids (MLVSSs) for endogenous respiration ($b^{\prime }$) conform to the empirical values typically used in domestic sewage treatment; parameters such as the oxygen transfer efficiency of fine bubble diffusers, the oxygen content in air, and the water temperature remain constant; and the MLVSS concentration is approximately the same in each compartment. For plug flow reactors (PFRs) and continuous stirred-tank reactors (CSTRs) following first-order reaction kinetics, the relationships between influent and effluent concentrations are presented by Equations (4) and (5), respectively [35].

(4) $$C=C_{0}\exp\left(-k\Theta \right),$$

(5) $$C=\frac{C_{0}}{1+\Theta k},$$

where C is the effluent BOD concentration (mg/L), C_0_ is the influent BOD concentration (mg/L), k is the first-order reaction rate constant (min^−1^), and Θ is the mean residence time (min).

Domestic sewage has a low organic (BOD) concentration, and the degradation process follows first-order reaction kinetics [36]. The effluent concentration formula for a single compartment under mixed flow pattern is presented as Equation (6).

(6) $$\;C=C_{0}\left[\frac{\alpha }{1+\Theta k}+\left(1\;-\;\alpha \right)\exp\left(-\Theta k\right)\right],$$

Define M as the attenuation coefficient of organic matter (BOD) concentration in a single compartment under mixed flow conditions:

(7) $$M\;=\;\left[\frac{\alpha }{1+\Theta k}\;+\;\left(1\;-\;\alpha \right)\exp\left(-\Theta k\right)\right],$$

where α is the fraction of completely mixed flow in a single compartment (%).

The effluent of the previous compartment serves as the influent of the next compartment. With an increase in the number of stages in series, the effluent concentration decays with the number of stages n following a M^n^ relationship, which is consistent with the pollutant degradation kinetics. Substituting these values into Equation (6) and performing iteration yields the effluent concentration of the reactant in the n-th compartment as shown in Equation (8).

(8) $$C_{n}={M^{n}\;C}_{0},$$

The pollutant removed in each stage is defined as ΔC_n_ = C_n−1_ – C_n_, which can be combined with Equation (8) to obtain:

(9) $$\Delta C_{n}={M^{n-1}(1-M)C}_{0},$$

The oxygen demand of the aeration tank includes the oxygen required for BOD degradation and endogenous respiration of activated sludge [37]. The oxygen demand R_n_ of the n-th compartment can be obtained as shown in Equation (10).

(10) $$R_{n}=a^{\prime }Q\Delta C_{n}+b^{\prime }VX_{v},$$

where R_n_ is the oxygen demand of the n-th compartment (kg O_2_/d), a′ is the oxygen required for metabolizing per kilogram of BOD (kg O_2_/kg BOD), Q is the wastewater flow rate (m^3^/d), b′ is the oxygen required for endogenous respiration of per kilogram of mixed liquor volatile suspended solids (MLVSSs) (kg O_2_/(kg MLVSS·d)), V is the volume of a single compartment (m^3^), X_v_ is the MLVSS concentration in the aeration tank (mg/L).

Combining Equations (9) and (10), the mathematical model for the oxygen demand in each compartment is obtained as follows:

(11) $$R_{n}=a^{\prime }Q{M^{n-1}(1-M)C}_{0}+b^{\prime }VX_{v},$$

All model input parameters were derived from measured operational data and the classical empirical coefficients recommended by the International Water Association (IWA). The first-order reaction rate constant k measured in experiments was 0.0113 min^−1^, the proportion of completely mixed flow α in a single compartment was 85.42%, and the mean residence time Θ of a single compartment was 155.34 min. As the substrate treated in this study was domestic sewage, the coefficients a′ and b′ were taken as 0.5 and 0.1, respectively [38]. In the experiment, Q was 0.1375 m^3^/d, V was 0.044 m^3^, and C_0_ was 114.73 mg/L. Substituting these values into Equation (11), along with an atmospheric oxygen content of approximately 21% [39], the theoretical aeration demand for each compartment was determined. The aeration rates for the first, second, third, and fourth compartments of the main reaction zone in the MCFCASS reactor were 4.95 L/min, 4.02 L/min, 3.22 L/min, and 2.68 L/min, respectively, corresponding to a ratio of 1.85:1.5:1.2:1.

The experiments under uniform aeration were completed before the theoretical derivation, and the total aeration rate was 13.33 L/min. To ensure a consistent total aeration rate between tapered and uniform aeration modes and to avoid interference from total aeration rate differences, the total aeration rate after redistribution remained at 13.33 L/min. Accordingly, based on the ratio of theoretical aeration rates for each compartment (i.e., 1.85:1.5:1.2:1), the aeration rates for the first, second, third, and fourth compartments of the main reaction zone under tapered aeration were determined as 4.43 L/min, 3.60 L/min, 2.90 L/min, and 2.40 L/min, respectively.

### 3.2. Pollutant Removal Performance Before and After Aeration Rate Redistribution

The MCFCASS reactor was started up using the inoculated biofilm method. Biofilm colonization was considered complete when both COD and NH_4_^+^-N removal efficiencies exceeded 80% and remained stable for approximately one week. Microscopic observation of the carriers revealed the presence of protozoa such as Vorticella and rotifers, and the biofilm thickness was observed to be approximately 1 mm. The total start-up period of the reactor in this study was 42 days, during which no sludge was discharged.

**Figure 2:**
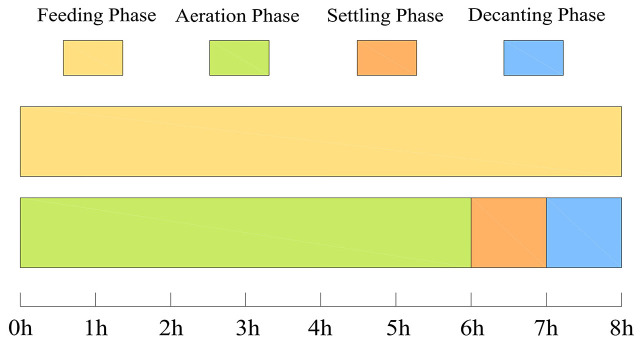
Operational procedure diagram of the MCFCASS reactor.

The MCFCASS reactor operated in continuous-flow mode with an operating cycle of 8 h, consisting of 6 h aeration, 1 h settling, and 1 h decanting, as illustrated in Figure 2. The MCFCASS reactor operated at an influent flow rate of 8.25 L/h, with a corresponding volumetric exchange ratio of 30%, a recycle ratio of 100%, and a sludge retention time (SRT) of 20 days. The influent entered the pre-reaction zone and then flowed in an S-shaped pattern through each compartment via the flow holes on the baffles. Under uniform aeration, the total aeration rate in the main reaction zone of the MCFCASS reactor was 13.33 L/min, with an identical aeration rate of 3.33 L/min for each compartment. After aeration rate redistribution, tapered aeration along the flow direction was adopted in the MCFCASS reactor. The total aeration rate of the main reaction zone was kept constant at 13.33 L/min. According to the optimized results from the mathematical model, the aeration rates of the first, second, third, and fourth compartments in the main reaction zone were adjusted to 4.43 L/min, 3.60 L/min, 2.90 L/min and 2.40 L/min, respectively.

Before comparing the pollutant removal performance of the MCFCASS reactor under the two aeration modes, the system was required to achieve steady-state operation. The steady-state criterion adopted in this study was as follows: the reactor operated continuously and stably for more than seven days, during which the daily fluctuations in COD, NH_4_^+^-N, and TN removal efficiencies were all within three percentage points, and no significant upward or downward trends were observed. Each water sample was measured in triplicate. Due to laboratory constraints, a single MCFCASS reactor initially operated under uniform aeration. After aeration rate redistribution, the reactor operated in tapered aeration mode. During the stable operation period under uniform aeration, the average mixed liquor suspended solid (MLSS) concentrations in the first and fourth compartments of the main reaction zone were 3513 mg/L and 3370 mg/L, respectively; during the stable operation period under tapered aeration, the average MLSS concentrations in the first and fourth compartments of the main reaction zone were 3720 mg/L and 3189 mg/L, respectively.

#### 3.2.1. Nitrogen Removal Performance Before and After Aeration Rate Redistribution

**Figure 3:**
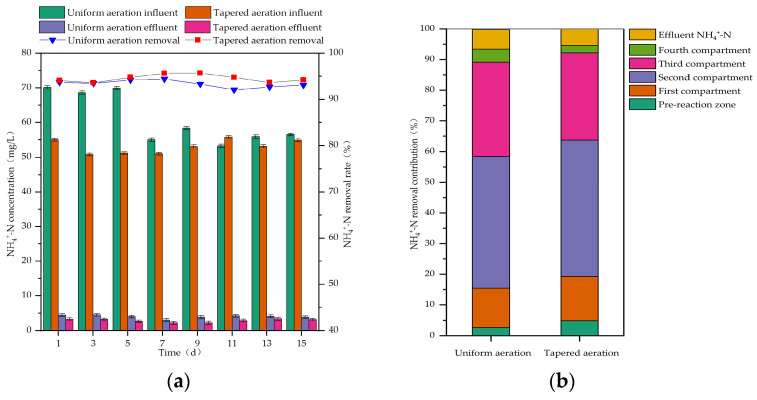
NH_4_^+^-N removal performance of the reactor before and after aeration rate redistribution: (**a**) Influent and effluent concentrations and removal rate of NH_4_^+^-N; (**b**) NH_4_^+^-N removal contribution rate of each compartment.

**Figure 4:**
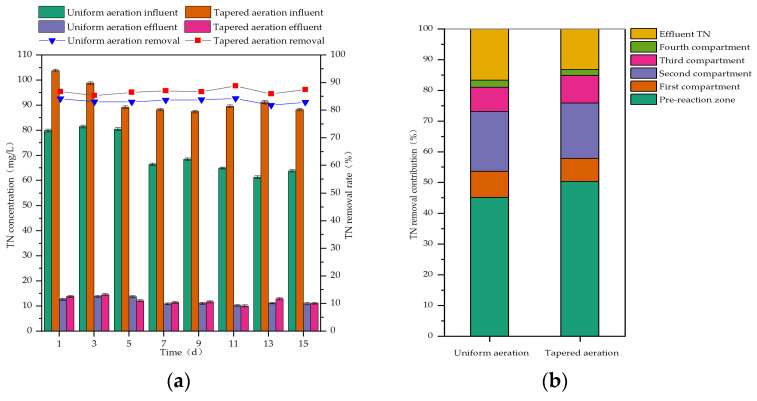
TN removal performance of the reactor before and after aeration rate redistribution: (**a**) Influent and effluent concentrations and removal rate of TN; (**b**) TN removal contribution rate of each compartment.

During uniform aeration, the influent NH_4_^+^-N concentration ranged from 53.27 to 70.12 mg/L, and the influent TN concentration ranged from 61.25 to 81.46 mg/L. During tapered aeration, the influent NH_4_^+^-N concentration ranged from 50.81 to 55.80 mg/L, and the influent TN concentration ranged from 87.41 to 103.79 mg/L. The removal efficiencies of NH_4_^+^-N and TN in the MCFCASS reactor before and after aeration rate redistribution, as well as the contribution of each compartment to NH_4_^+^-N and TN removal, are shown in Figure 3 and Figure 4.

As shown in Figure 3a, after aeration rate redistribution, the average effluent concentration of NH_4_^+^-N in the MCFCASS reactor decreased from 4.02 mg/L to 2.85 mg/L, and the average removal efficiency increased from 93.39% to 94.57%. As shown in Figure 3b, the average NH_4_^+^-N removal contribution of the pre-reaction zone increased from 2.65% to 4.87%. In the main reaction zone, the average NH_4_^+^-N removal contributions of the first and second compartments rose from 12.78% to 14.38% and from 42.99% to 44.45%, respectively; while those of the third and fourth compartments decreased, from 30.72% to 28.48% and from 4.25% to 2.39%, respectively. Before aeration rate redistribution, insufficient aeration in the first compartment of the main reaction zone led to competition for dissolved oxygen between heterotrophic and nitrifying bacteria, which inhibited the activity of nitrifying bacteria to some extent [40], resulting in a relatively low NH_4_^+^-N removal contribution in this compartment. After aeration rate redistribution, the increased aeration rates in the first and second compartments of the main reaction zone may have enhanced nitrifying bacterial activity and promoted mass transfer, thereby slightly improving nitrification efficiency [41]. Consequently, the NH_4_^+^-N removal contributions of the first and second compartments increased slightly. Similarly, Castagnoli et al. also observed an increasing trend of NH_4_^+^-N removal efficiency with increasing aeration rate [42]. Meanwhile, the aeration rates in the subsequent compartments of the MCFCASS reactor were reduced, leading to a corresponding decrease in their NH_4_^+^-N removal contributions.

As shown in Figure 4a, during the stable operation period of uniform aeration, the average influent and effluent TN concentrations were 70.82 mg/L and 11.79 mg/L, respectively. After aeration rate redistribution, during the stable operation period of tapered aeration, the average influent and effluent TN concentrations were 92.05 mg/L and 12.17 mg/L, respectively. The average TN removal efficiency of the MCFCASS reactor increased from 83.34% to 86.80%. As shown in Figure 4b, the average TN removal contribution of the pre-reaction zone rose from 45.10% to 50.26%, and that of the third compartment in the main reaction zone increased from 7.92% to 8.94%. In contrast, the average TN removal contributions of the first, second, and fourth compartments in the main reaction zone decreased from 8.49% to 7.54%, from 19.51% to 18.12%, and from 2.32% to 1.94%, respectively. After aeration rate redistribution, the MCFCASS reactor maintained favorable nitrogen removal performance under elevated influent TN loading, complying with the Grade I-A (15.00 mg/L) of GB 18918-2002 [43]. The aeration rate in the fourth compartment of the main reaction zone in the MCFCASS reactor was reduced from 3.33 L/min to 2.40 L/min. Correspondingly, the average DO concentration in this compartment decreased from 4.13 mg/L to 3.32 mg/L, leading to a lower DO level in the mixed liquor recirculated to the pre-reaction zone. The average DO concentration in the pre-reaction zone decreased from 0.54 mg/L to 0.43 mg/L. During the stable operation periods under uniform and tapered aeration, MPN counts showed that the number of denitrifying bacteria in the pre-reaction zone of the MCFCASS reactor was 5.2 × 10^6^ cfu/mL (colony-forming units per milliliter) under tapered aeration, which was higher than the value of 2.5 × 10^6^ cfu/mL under uniform aeration. This increase was consistent with the observed rise in the average TN removal efficiency. These results suggest that tapered aeration could, to some extent, mitigate the interference of dissolved oxygen carried by the return mixed liquor with the anoxic denitrification environment in the pre-reaction zone, thereby contributing to the improved TN removal performance [22]. Meanwhile, after aeration rate redistribution, nitrification in the main reaction zone was somewhat enhanced, and the nitrate (NO_3_^−^) produced was recirculated with the mixed liquor to the pre-reaction zone as an electron acceptor. This contributed positively to the denitrification performance in the pre-reaction zone [44].

#### 3.2.2. Organic Matter Removal Performance Before and After Aeration Redistribution

**Figure 5:**
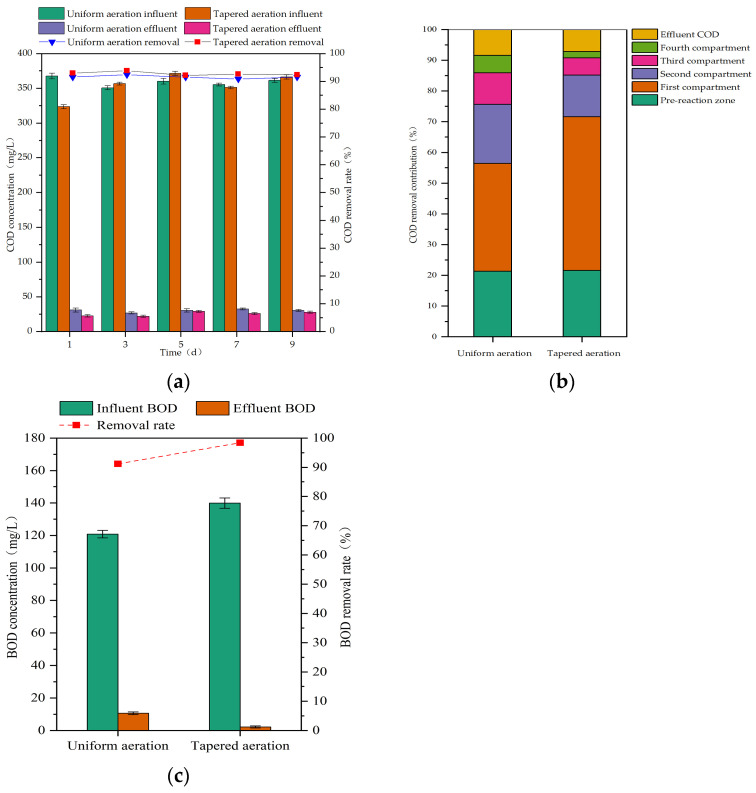
Organic matter removal performance of the reactor before and after aeration rate redistribution: (**a**) Influent and effluent concentrations and removal rate of COD; (**b**) COD removal contribution rate of each compartment; (**c**) Average influent and effluent concentrations and average removal rate of BOD.

During uniform aeration, the influent COD concentration ranged from 350.73 to 367.60 mg/L, and the influent BOD concentration ranged from 114.86 to 126.95 mg/L. During tapered aeration, the influent COD concentration ranged from 323.57 to 371.43 mg/L, and the influent BOD concentration ranged from 130.07 to 150.54 mg/L. The BOD removal rate, COD removal rate, and compartmental COD removal contribution of the MCFCASS reactor before and after aeration rate redistribution are shown in Figure 5.

As shown in Figure 5a, after aeration rate redistribution, the average effluent COD concentration of the MCFCASS reactor decreased from 30.26 mg/L to 25.53 mg/L, and the average removal efficiency increased from 91.58% to 92.98%. As shown in Figure 5b, the average COD removal contribution of the pre-reaction zone increased slightly from 21.34% to 21.75%, while that of the first compartment in the main reaction zone rose substantially from 35.08% to 47.64%. In contrast, the average COD removal contributions of the second, third, and fourth compartments in the main reaction zone decreased from 19.21%, 10.27%, and 5.67% to 14.99%, 6.21%, and 2.59%, respectively. As shown in Figure 5c, the average effluent BOD concentration of the MCFCASS reactor decreased from 10.62 mg/L to 2.21 mg/L, and the average removal rate increased from 91.21% to 98.42%. After aeration rate redistribution, the BOD removal rate of the MCFCASS reactor was improved, and the first compartment of the main reaction zone became the dominant region for COD removal. This may be attributed to the increased aeration rate in the front part of the main reaction zone under tapered aeration, which enhanced microbial activity and substrate utilization in the first compartment [45,46]. Since most organic matter was removed in the first compartment, the organic substrate concentration in the subsequent compartments decreased, which may be favorable for the proliferation of nitrifying bacteria [47].

### 3.3. Microbial Community Structure Before and After Aeration Rate Redistribution

**Figure 6:**
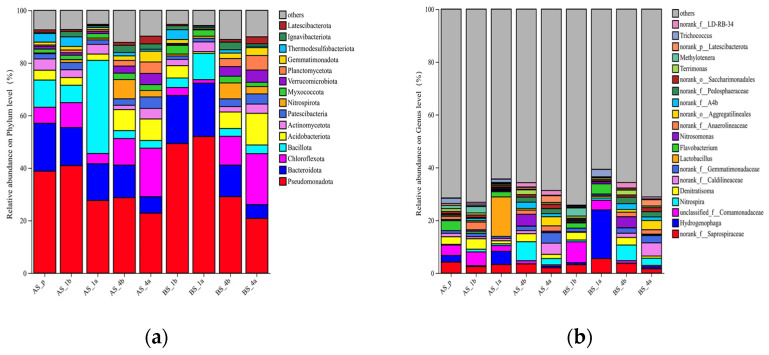
Microbial community structure before and after aeration rate redistribution: (**a**) the phylum level; (**b**) the genus level. Before aeration rate redistribution, suspended sludge samples from compartments 1 and 4 of the main reaction zone were labeled AS_1b and AS_4b, and biofilm samples as BS_1b and BS_4b. After redistribution, the sample from the pre-reaction zone was denoted AS_p; suspended sludge samples from compartments 1 and 4 were labeled AS_1a and AS_4a, and biofilm samples as BS_1a and BS_4a.

As shown in Figure 6a,b, after aeration rate redistribution, the relative abundances of Pseudomonadota, Bacteroidota, and Bacillota at the phylum level in the pre-reaction zone of the MCFCASS reactor were 39.17%, 17.78%, and 10.33%, respectively. The abundances of these phyla were similar to or higher than those in the anoxic zone of some similar systems, as shown in Table 2. As reported in previous studies, Pseudomonadota may participate in biological nitrogen removal and organic matter degradation in wastewater treatment processes [48]; Bacteroidota may promote the utilization of nitrogen-containing organics, biotransformation of steroids, and hydrolysis of macromolecular substances [49]; Bacillota may participate in the catabolism of organic intermediates such as volatile fatty acids and convert complex organics into small-molecule substances via hydrolytic acidification [50]. At the genus level in the pre-reaction zone, the abundance of *norank_f__Saprospiraceae*, potentially with organic degradation capability, was 4.18%; the abundances of the denitrifying genera *unclassified_f__Comamonadaceae* and *Denitratisoma* were 3.96% and 2.89%, respectively; the abundance of *Flavobacterium* was 3.78%, and this genus may include strains with denitrifying and phosphate accumulating capabilities [51], which can participate in the nitrogen and phosphorus removal process.

After aeration rate redistribution, the abundance of Pseudomonadota in the biofilm of the first compartment in the main reaction zone increased from 50.16% to 52.20%. Among them, the abundances of Nitrosomonas and Nitrospira in the biofilm increased from 0.34% to 0.77% and from 0.69% to 0.83%, respectively. The abundances of some nitrifying genera in the first compartment of the main reaction zone increased slightly, which is consistent with the slightly increased contribution rate of NH_4_^+^-N removal in this compartment shown in Figure 3.

The abundance of Bacillota in the biofilm of the first compartment increased from 3.64% to 10.03%, while in the suspended sludge it rose markedly from 6.61% to 35.46%. Among these, *Lactobacillus* accounted for 14.48%. This genus may ferment soluble organic matter to produce volatile fatty acids (VFAs), which may provide potential carbon sources for denitrification in the pre-reaction zone through mixed liquor recirculation [56]. Meanwhile, the abundance of Bacteroidota in the biofilm increased from 18.31% to 20.28%. The abundances of *norank_f__Saprospiraceae* in the biofilm and suspended sludge increased from 3.18% to 5.48% and from 2.45% to 3.25%, respectively. This genus, as a hydrolytic bacterium, may degrade particulate organic matter to produce small-molecule carbon sources [57]. The abundances of some organic-degrading genera in the first compartment of the main reaction zone increased, which is consistent with the increasing trend of COD removal contribution in this compartment shown in Figure 5.

After aeration rate redistribution, the aeration rate in the fourth compartment of the main reaction zone was reduced. The abundance of Bacteroidota in the biofilm decreased from 11.89% to 5.34%, the abundance of Pseudomonadota decreased from 29.17% to 20.96%, and the abundance of *Nitrospirota* in the suspended sludge decreased from 5.92% to 2.72%. The abundances of some phyla in the fourth compartment of the main reaction zone decreased, which is consistent with the reduced removal contributions of NH_4_^+^-N and COD in this compartment shown in Figure 3 and Figure 5. The organic concentration in the fourth compartment was relatively low, while the abundance of Chloroflexota in the biofilm increased from 10.95% to 19.41%. This may be because some members of the phylum Chloroflexota are capable of accumulating intracellular glycogen, which can be degraded under carbon-limited conditions [58].

Previous studies have shown that aeration rate can regulate the DO concentration in a reactor, and changes in DO concentration drive adaptive shifts in microbial community structure [59], which is similar to the results obtained in this study. After aeration rate redistribution, the aeration rate was increased in the first compartment and decreased in the fourth compartment of the main reaction zone. Microbial analysis indicated that in the first compartment, the abundances of some organic-degrading bacteria increased and those of some nitrifying genera increased slightly; in the fourth compartment, the abundances of related functional bacteria decreased. These changes were consistent with the observed trends in the contribution rates of NH_4_^+^-N, TN, and COD removal in the first and fourth compartments of the main reaction zone.

**Figure 7:**
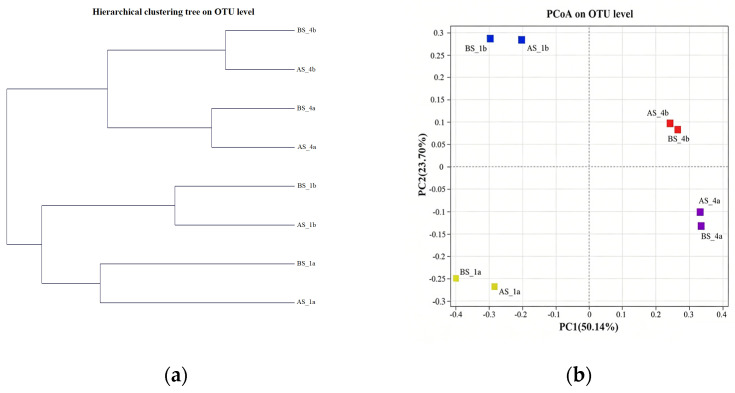
Hierarchical clustering and principal coordinates analysis plot of suspended sludge and biofilm samples from the first and fourth compartments of the main reaction zone before and after aeration rate redistribution: (**a**) Hierarchical clustering tree at the OTU level; (**b**) PCoA plot at the OTU level.

As shown in Figure 7a, the hierarchical clustering tree at the OTU level divided all samples into two main branches. One branch included all samples from the first compartment (BS_1b, AS_1b, BS_1a, AS_1a), and the other branch included all samples from the fourth compartment (BS_4b, AS_4b, BS_4a, AS_4a). This result indicates that distinct differences in microbial community structure existed between the first and fourth compartments both before and after aeration rate redistribution, and that the inter-compartment differences were greater than those caused by changes in aeration rate within the same compartment. Furthermore, within each compartment, samples collected before and after aeration rate redistribution formed separate smaller subclusters, suggesting that the impact of aeration adjustment on the microbial communities of suspended sludge and biofilm within the same compartment was consistent.

As shown in Figure 7b, principal coordinate analysis (PCoA) revealed that PC1 explained 50.14% of the community variation, PC2 explained 23.70%, and the two axes together explained 73.84% of the total variation, effectively reflecting differences in community structure among samples. The samples from the first compartment were distributed on the positive side of PC1, while those from the fourth compartment were distributed on the negative side, further indicating that compartment location was the most important factor shaping microbial community structure. Samples collected before aeration rate redistribution were located on the positive side of PC2, and those collected after redistribution on the negative side, demonstrating that the microbial community structure underwent adaptive changes in response to aeration rate adjustment in each compartment.

## 4. Conclusions

In this study, a novel multi-compartment fixed biofilm cyclic activated sludge system (MCFCASS) was constructed. By dividing the main reaction zone into four serially connected compartments, the plug–flow ratio of the system was increased from 18.75% to 31.25%. Shield-shaped fiber packing was suspended in each compartment. After aeration rate redistribution, the TN removal efficiency of the MCFCASS reactor increased from 83.34% to 86.80%.

Based on the hydraulic flow pattern experiments and the relevant assumptions, a mathematical model for aeration rate redistribution was established. Under the tapered aeration mode, the aeration rate in each compartment better matched the oxygen demand, thereby improving improved oxygen utilization efficiency. Meanwhile, the DO concentration in the recirculated mixed liquor decreased, potentially mitigating disturbance to the anoxic denitrification environment in the pre-reaction zone. After aeration rate redistribution, the microbial community structure in the first and fourth compartments of the main reaction zone of the MCFCASS reactor underwent adaptive changes in response to the adjustment of aeration rates, consistent with the observed trends in the pollutant removal contributions of these compartments. Under fluctuating influent TN loading conditions, the MCFCASS reactor operating in tapered aeration mode exhibited satisfactory nitrogen removal performance. These laboratory-scale results of aeration rate redistribution may provide a preliminary reference for aeration control in similar nitrogen removal processes.

## Figures and Tables

**Table 1:** Fractions of Plug Flow and Completely Mixed Flow in CASS and MCFCASS Reactors.

Reactor	t_c_ (min)	f_1_ (%)	1 – f_1_ (%)
CASS	225	81.25%	18.75%
MCFCASS	375	68.75%	31.25%
Single compartment in the MCFCASS reactor	35	85.42%	14.58%

**Table 2:** Comparison of dominant phyla abundance in the pre-reaction zone of the MCFCASS reactor.

Sampling Area	Pseudomonadota Abundance	Bacteroidota Abundance	Bacillota Abundance	Reference
The anoxic zone sludge of A/O system	36.09% ^1^	6.68% ^2^	2.35% ^2^	[52,53]
The anoxic zone sludge of A/A/O system	31.84% ^3^ (32.87%) ^4^	6.68% ^3^ (7.90%) ^4^	2.35% ^3^ (11.95%) ^4^	[54,55]
The anoxic zone sludge of the CASS	33.70%	13.30%	3.20%	[18]
The pre-reaction zone sludge of the MCFCASS reactor	39.17%	17.78%	10.33%	This study

^1^ indicates data cited from reference [52], ^2^ indicates data cited from reference [53], ^3^ indicates data cited from reference [54], ^4^ indicates data cited from reference [55].

## Data Availability

The original contributions presented in this study are included in the article. Further inquiries can be directed to the corresponding author.

## Abbreviations

The following abbreviations are used in this manuscript:

A/O	Anoxic/Oxic
A/A/O	Anaerobic/Anoxic/Oxic
BOD	Biochemical Oxygen Demand
CASS	Cyclic Activated Sludge System
COD	Chemical Oxygen Demand
CSTR	Continuous Stirred-Tank Reactor
DO	Dissolved Oxygen
MCFCASS	Multi-Compartment Fixed-Biofilm Cyclic Activated Sludge System
MLE	Modified Ludzack–Ettinger
MLSS	Mixed Liquor Suspended Solids
MLVSS	Mixed Liquor Volatile Suspended Solids
MPN	Most Probable Number
PCoA	Principal Coordinate Analysis
PFR	Plug Flow Reactor
PVA	Polyvinyl Alcohol
RTD	Residence Time Distribution
SBR	Sequencing Batch Reactor
SRT	Sludge Retention Time
TN	Total Nitrogen
UV	Ultraviolet
